# Longitudinal assessment of bleomycin-induced lung fibrosis by Micro-CT correlates with histological evaluation in mice

**DOI:** 10.1186/s40248-017-0089-0

**Published:** 2017-04-10

**Authors:** Francesca Ruscitti, Francesca Ravanetti, Jeroen Essers, Yanto Ridwan, Sasha Belenkov, Wim Vos, Francisca Ferreira, Alex KleinJan, Paula van Heijningen, Cedric Van Holsbeke, Antonio Cacchioli, Gino Villetti, Franco Fabio Stellari

**Affiliations:** 1 Chiesi S.p.A., Pre-Clinical R & D, Parma, Italy; 2 Dipartimento di Scienze Medico Veterinarie, Università di Parma, Parma, Italy; 3 Department of Molecular Genetics, Vascular Surgery, and Radiation Oncology, Erasmus MC, Rotterdam, The Netherlands; 4 PerkinElmer, Inc, Waltham, MA USA; 5 Fluidda NV, Kontich, Belgium; 6 Department of Pulmonary Medicine Erasmus MC, Rotterdam, The Netherlands; 7 Chiesi Farmaceutici, Pharmacology & Toxicology Department Corporate Pre-Clinical R & D, Largo Belloli, 11/A, Parma, 43122 Italy

**Keywords:** Bleomycin, Lung fibrosis, Micro-CT, Mice

## Abstract

**Background:**

The intratracheal instillation of bleomycin in mice induces early damage to alveolar epithelial cells and development of inflammation followed by fibrotic tissue changes and represents the most widely used model of pulmonary fibrosis to investigate human IPF.

Histopathology is the gold standard for assessing lung fibrosis in rodents, however it precludes repeated and longitudinal measurements of disease progression and does not provide information on spatial and temporal distribution of tissue damage.

Here we investigated the use of the Micro-CT technique to allow the evaluation of disease onset and progression at different time-points in the mouse bleomycin model of lung fibrosis. Micro-CT was throughout coupled with histological analysis for the validation of the imaging results.

**Methods:**

In bleomycin-instilled and control mice, airways and lung morphology changes were assessed and reconstructed at baseline, 7, 14 and 21 days post-treatment based on Micro-CT images. Ashcroft score, percentage of collagen content and percentage of alveolar air area were detected on lung slides processed by histology and subsequently compared with Micro-CT parameters.

**Results:**

Extent (%) of fibrosis measured by Micro-CT correlated with Ashcroft score, the percentage of collagen content and the percentage of alveolar air area (*r* ^*2*^ = 0.91; 0.77; 0.94, respectively). Distal airway radius also correlated with the Ashcroft score, the collagen content and alveolar air area percentage (*r* ^*2*^ = 0.89; 0.78; 0.98, respectively).

**Conclusions:**

Micro-CT data were in good agreement with histological read-outs as micro-CT was able to quantify effectively and non-invasively disease progression longitudinally and to reduce the variability and number of animals used to assess the damage. This suggests that this technique is a powerful tool for understanding experimental pulmonary fibrosis and that its use could translate into a more efficient drug discovery process, also helping to fill the gap between preclinical setting and clinical practice.

## Background

Idiopathic pulmonary fibrosis (IPF) is a chronic, progressively fatal lung disease characterized by fibroblast proliferation and extracellular matrix remodeling. IPF etiology is unknown and possesses the worst prognosis among all interstitial lung diseases, with a median survival of 2–4 years after diagnosis [1].

The pathogenesis of IPF is not fully understood. It has been hypothesized that multiple cycles of lung injuries lead to destruction of epithelial alveolar cells that in turn cause the migration, proliferation and activation of mesenchymal cells and the exaggerated accumulation of fibroblasts and myofibroblasts. This induces an excessive collagen deposition within the lung interstitium and alveolar space, mirroring abnormal wound repair [1, 2]. In humans, IPF manifests histopathologically as usual interstitial pneumonia (UIP) and as subpleural and basal predominant reticulation with honeycombing on high-resolution computed tomography (HRCT) of the chest [3].

Bleomycin is a glycosylated linear non-ribosomal peptide antibiotic produced by the bacterium Streptomyces verticillus. Bleomycin has potent tumor killing properties, which have given it an important role in cancer chemotherapy for curable diseases such as germinative tumors and Hodgkin’s lymphoma. However, the major limitation of bleomycin therapy is pulmonary toxicity which can be life threatening in up to 10% of the patients receiving the drug [4–6].

Based on these evidences, bleomycin is the most widely used drug for the induction of experimental pulmonary fibrosis. The bleomycin model has many advantages: it is easy to perform, widely accessible and highly reproducible [7, 8].

Whilst lung bleomycin administration is considered the easiest and the most reproducible way to induce consistent lung fibrosis in rodents, concerns remain with regard to the temporal aspects of the model and the translation of the results to the clinic. The bleomycin model has two distinct phases: the inflammatory stage that develops within the first 2 weeks after the injury, which then subsides to the fibrotic phase. It is now evident that targeting mechanisms occurring during the inflammatory phase are not likely to translate into a clinical benefit and that in order to determine new drugs efficacy their testing must be performed during the fibrotic phase [9].

The conventional assessment tools used in the bleomycin model [9] are terminal and primarily based on labor-intensive histology and quantitative assessment of biochemical biomarkers (i.e. hydroxyproline levels and lung collagen content) [10–12]. These parameters are known to be highly variable among animals and represent only a snapshot at a given time-point of a far more complex and dynamic biological process. Neither histopathology nor biochemical analysis provide information about the temporal and spatial distribution of the fibrotic lesion over the course of the study. Moreover, the tendency for a spontaneous resolution of the fibrotic lesion, its patchy distribution with some areas of the lung unaffected, and possible sampling errors with regard to the selection and the number of fields to be analyzed, are additional shortcomings that hamper successful translation of the results to the clinic.

Recently, non-invasive Magnetic Resonance (MRI), Micro-Computer Tomography (Micro-CT) and optical techniques have been applied to longitudinal monitoring of airway remodeling and inflammation in murine models [13–17].

In particular, lung Micro-CT providing high air-tissue contrast is being increasingly used in pulmonary research for the investigation of lung fibrosis in experimental animals. Initial evidence suggests that by using this imaging modality it is possible to quantify early pathological changes associated with pulmonary fibrosis that correlate to histopathological findings [18–20].

Indeed, serial Micro-CT based analysis as compared with classical histological approaches results in quantitative datasets that should allow for longitudinal assessment, comparison among different treatment groups including the effect of therapeutic intervention and detailed tomographic information documenting the extent of the disease in the individual animal.

Macro-CT and Micro-CT based functional respiratory imaging (FRI) has demonstrated to be a powerful tool to look at disease progression in IPF. Using FRI, different studies have been performed in both human subjects and animal models to assess the response to drug and intervention therapy [21–23], to assist in better diagnosis [24] and to study regional variations in lung structure and function [25].

In this study, we intended to quantify longitudinally lung fibrosis progression and airway radius changes by using a semi-automated algorithm at different time-points after bleomycin administration in mice starting from baseline and at days 7, 14 and 21. Furthermore, complementary histological measurements were performed at all time-points to validate the outcome of the serial Micro-CT investigation.

## Methods

### Experimental set up

Sixty mice were randomly assigned to receive either saline or bleomycin (dose) and were double instilled intratracheally on day 0 and 4. Lungs were harvested in 5 animals per group for histological analysis at 7, 14 and 21 days after the first treatment. Parallel groups of 5 animals for each treatment arm were imaged with micro-CT at the same time-points and the lungs harvested soon after the scanning to assess the correlation between micro-CT with histological data.

### Experimental animals

C57Bl/6 female mice, 8–9 weeks supplied by San Pietro NatisoneHorst, The Netherlands (UD), were used. Prior to use, animals were acclimatized for at least 7 days to the local vivarium conditions (room temperature: 20–24 °C; relative humidity: 40–70%; 12-h light–dark cycle), having free access to standard rodent chow and softened tap water. All animal experiments described herein were approved by the intramural animal-welfare committee for animal experimentation of Chiesi Farmaceutici and ERASMUS MC under protocol number: EMC 3349 (138-14-07) comply with the European Directive 2010/63 UE, Italian D.Lgs 26/2014 and the revised “Guide for the Care and Use of Laboratory Animals” [10].

### Bleomycin administration

**Fig. 1:**
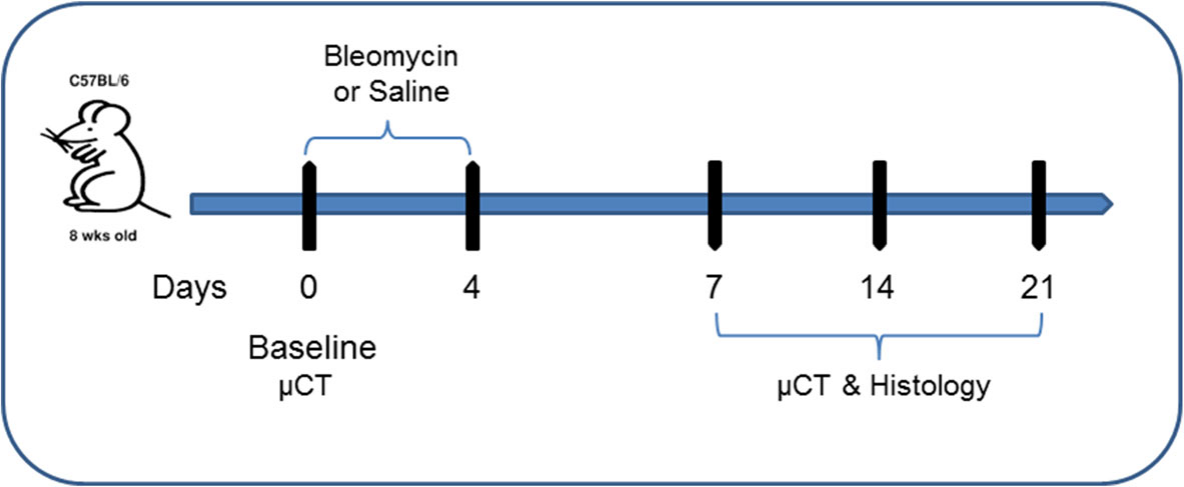
Experimental timeline for bleomycin-induced lung mouse fibrosis. Sixty C57Bl/6 female were randomly assigned to receive either saline or bleomycin (dose) and were double instilled intratracheally on day 0 and 4. Lungs were harvested in 5 animals per group for histological analysis at 7, 14 and 21 days after the first treatment. Parallel groups of 5 animals for each treatment arm were imaged with micro-CT at the same time-points and the lungs harvested soon after the scanning to assess the correlation between micro-CT with histological data

For intratracheal administrations, 50 μl/mouse either saline or bleomycin sulphate from Streptomyces Verticillus (Sigma B2434) (1 mg/Kg) dissolved in saline were instilled into the tracheal lumen through a cannula under isoflurane (2.5% in oxygen) anaesthesia. The procedure was performed using a small laryngoscope (Penn-Century Inc., Philadelphia, U.S.A.) to visualise the epiglottis and ensure a good positioning of the cannula into the tracheal lumen. Bleomycin or saline were injected at day 0 and at day 4 (Fig. 1).

### Micro-CT scanning

After mice were anaesthetized with isoflurane, the mouse lung imaging was performed with a Quantum FX Micro-CT scanner (PerkinElmer, Inc., Waltham, MA) using a cardio-respiratory gated technique as reported previously (PMID: 26967592). The X-ray system of the scanner uses a microfocus tube with a focal spot size of 5 μm at 4 W and produces X-rays in a cone-beam geometric formation. The detection system of the scanner is composed of an amorphous silicon digital X-ray plat panel which can acquire projection radiographs at 30 frames per second. The images were acquired with the X-ray tube set to 90 kVp, 160 μA and projection radiographs were taken throughout the 360° gantry rotation for a total scan time of 4.5 min. The entire set of projection radiographs was retrospectively gated using proprietary intrinsic image-based gating and then reconstructed at functional residual capacity (FRC) by a modified Feldkamp cone-beam filtered back-projection algorithm. The reconstructed volume was 20 × 20 × 20 mm and the voxel size was 40 × 40 × 40 μm.

### Image processing

#### Micro-CT data were analyzed by an independent company (FLUIDDA NV, Belgium)

Micro-CT scan data was converted into 3D models of airways, lung lobes and fibrosis using Mimics 15 (Materialise, Leuven, Belgium) a previously validated software package (Food and Drug Administration, K073468; Conformité Européenne certificate, BE 05/1191.CE.01). Airways and lungs were obtained based on thresholding segmentation. Lungs were split into lobes by identification of the fissure lines from the CT scan. We identified lobes as: Right Cranial Lobe (RCrL), Right Middle Lobe (RMdL), Right Caudal Lobe (RCdL), Right Accessory Lobe (RAcL) and Left Lung (LL) (Fig. 5b).

Airway models were split into trachea and airways leading to each previously defined lobe (Fig. 5a). Airway radius was given by the average airway lumen radius per zone. The FRI fibrosis parameter was obtained based on thresholding segmentation and consequent intersection with the lobe masks in order to obtain regional information. Since blood is not a natural contrast on CT images, if no contrast agent is added when performing the scan, no distinction can be made between blood vessels and fibrosis. For that reason, the FRI fibrosis parameter is often a combination of blood vessels and fibrosis.

### Histology

Mice were sacrificed and lungs were removed and inflated with a cannula through the trachea by gentle infusion with 0.6 ml of 10% neutral-buffered formalin. The lungs were then placed in a vial containing 10% formalin and fixed for 24 h. For histological assessment, the whole lungs were dehydrated in graded ethanol series, clarified in xylene and paraffin embedded.

Sections of 5 μm thick were cut with a rotary microtome (Reichert-Jung 1150/Autocut) in dorsal plane. The sections were Masson’s trichrome stained according to the manufacturer’s specifications (Histo-Line Laboratories). For analysis the whole-slide imaging were acquired by the NanoZoomer S-60 Digital slide scanner (Hamamatsu). Fibrotic lung injury was assessed morphologically by semi-quantitative and quantitative parameters as follow. Two independent pathologists with experience in animal models of lung fibrosis performed blind histological analysis of the specimens/slides.

#### Ashcroft score

Morphological changes in lung sections were graded semi-quantitatively according to the scale defined by Ashcroft [26] modified by Hṻbner et al. [27]. All the parenchyma in the lung sections were assessed by a system of 0–8 score. In every field (20× magnification microscopic field), the predominant degree of fibrosis was recorded as that occupying more than half of the field area. The final score was expressed as a mean of individual scores observed on all microscopic fields.

#### Collagen content

The degree of fibrosis was quantified by the NIS-AR image analysis software (Nikon). As ROIs three 10× magnification 1.1 × 0.9 mm, randomly selected were considered per each slide. By standardization of image contrast, brightness and colour threshold settings, the image analysis program was configured to detect areas of green-stained collagen within each ROI. The collagen content is expressed as a percentage of collagen deposition area (μm^2^) referred to the lung total area (μm^2^) within the ROI. Bronchi and blood vessels has been removed from the ROI area.

#### Alveolar air area

As an indirect parameter of fibrosis the alveolar air area was quantified. By mean of the same software and ROIs applied for the fibrosis fraction, the air area within the ROIs was detected using a white threshold. The Air Area is expressed as a percentage of area (μm^2^) occupied by air referred to the lung total area (μm^2^) within the ROI. Bronchi and blood vessels has been removed from the ROI area.

### Data analysis

Data are expressed as the mean and the standard error of the mean (s.e.m.). Statistical analysis was performed using one-way ANOVA followed by Dunnett’s t test for multiple comparison (PRISM Statistical software v 4.0.3). *p < *0.05*, **p < *0.01* was considered a level of statistical significance.

## Results

Development of pulmonary fibrosis induced by intratracheal administration of bleomycin in mice is often unpredictable. There is a high degree of variability among individual animals in the extent of fibrosis and the fibrosis often tends to resolve spontaneously if inhaled bleomycin is administered as single shot. This represents a real limitation for pharmacological studies due to the restricted time window available for testing new drugs as therapeutic intervention.

To overcome these issues, in the present study we have used a double instillation of bleomycin for inducing a more robust and reproducible lung fibrosis as detailed in the experimental set up and described in Fig. 1.

**Fig. 2:**
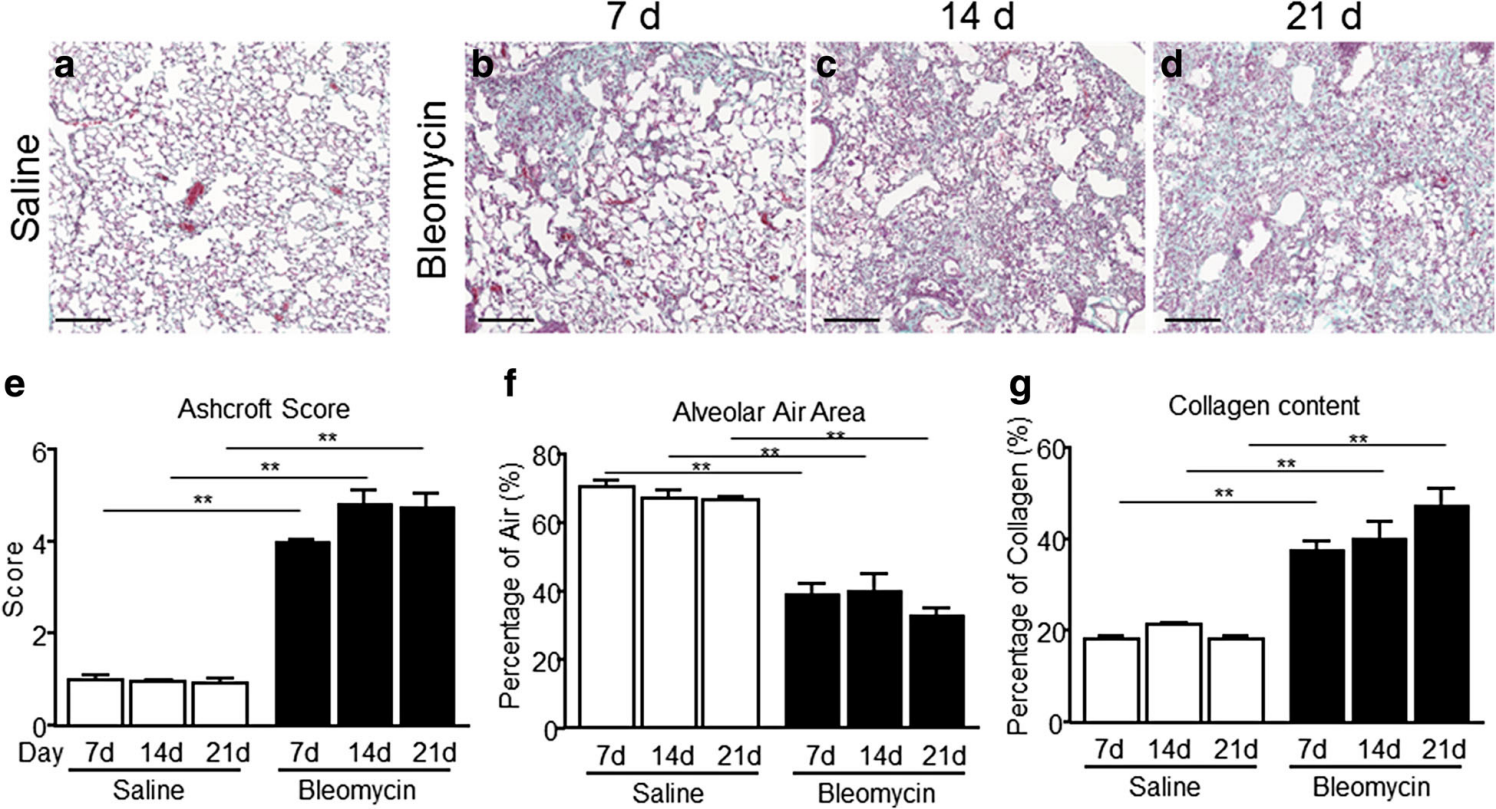
Quantitative histological analysis of lung fibrosis progression in bleomycin mouse model. Representative images of Trichromic Masson-staining of mice lung sections: of saline and bleomycin-treated mice over time. **a** saline, (**b**, **c** and **d**). Bleomycin treated mice at 7–14 and 21 respectively used for the quantification (10×). Bars: 200 μm. Time course of disease progression of saline and bleomicyn-treated mice at 7, 14 and 21 days: **e** Ashcroft’s score criteria, **f** Alveolar Air area percentage and **g** Collagen content percentage. The experiment was repeated three times and each point presents the mean ± s.e.m of 10 animals, for total of 60 mice per experimental session. Changes were compared to the saline group using one-way ANOVA followed by Dunnett’s t test for multiple comparison. *p < 0.05; **p < 0.01

Masson’s trichrome staining was used to stage the severity of the fibrosis by Ashcroft score and to quantify collagen content percentage and alveolar air area fraction percentage (Fig. 2e, f and g). Representative images of histological sections stained with Masson’s trichrome showing the fibrosis progression of saline and bleomycin-treated mice at the designated time-points are shown in Fig. 2a, b, c, and d.

Histopathological examination revealed that bleomycin induced a fibrotic pattern characterized by a patchy distribution of the fibrotic foci in the lung parenchyma that lead to a marked geographic heterogeneity in the distribution of fibrosis within the same lobe.

In the bleomycin-treated groups, the Ashcroft score was significantly increased for the whole duration of the study compared with the saline groups (Fig. 2e). Single fibrotic masses were evident starting from day 7 (Score 4) (Fig. 2b) and evolved into confluent conglomerates of substitutive collagen at day 14 (Score 4.83) (Fig. 2c). At day 21, the fibrotic score remained stable (Score 4.76) (Fig. 2d), and no changes in morphological distribution and appearance of fibrosis pattern were revealed. The saline-treated group, as expected, showed normal lung architecture (Score <1) at all points of observation with no prominent inflammation or fibrosis masses in the parenchyma (Fig. 2a and e).

Histomorphometric analysis of the alveolar air area in control animals revealed that this parameter was unchanged at all time-points, representing around 70% of total lung area. On the contrary, a significant decrease in the alveolar air area percentage was observed in bleomycin injured mice compared to control groups over the course of the study (Fig. 2f). Fully in agreement with the other two parameters measured, the collagen content percentage was significant increased over the time in bleomycin-treated mice compared with saline group (Fig. 2g). Indeed, the areas of fibrosis that stained positively for collagen were increased at day 7, peaking at day 14 and remained stable till the last point of observation.

**Fig. 3:**
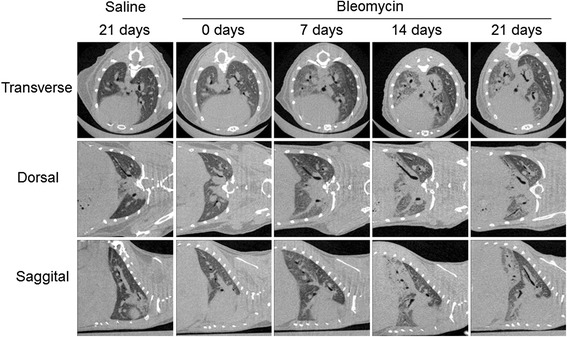
Serial Micro-CT imaging during bleomycin-induced lung fibrosis. 3D Micro-CT imaging of progressive pulmonary fibrosis in a representative saline and bleomycin-treated mouse over time. Mice were scanned weekly, from baseline to 21 days after induction

In Fig. 3, Micro-CT scan images from a 21 days saline-treated mouse and the same bleomycin-treated mice at baseline, 7, 14 and 21 days are shown, highlighting the capability of Micro-CT to visualize longitudinally the progressive anatomical changes of the lung architecture, well visible if compare to the saline-treated mice projections.

**Fig. 4:**
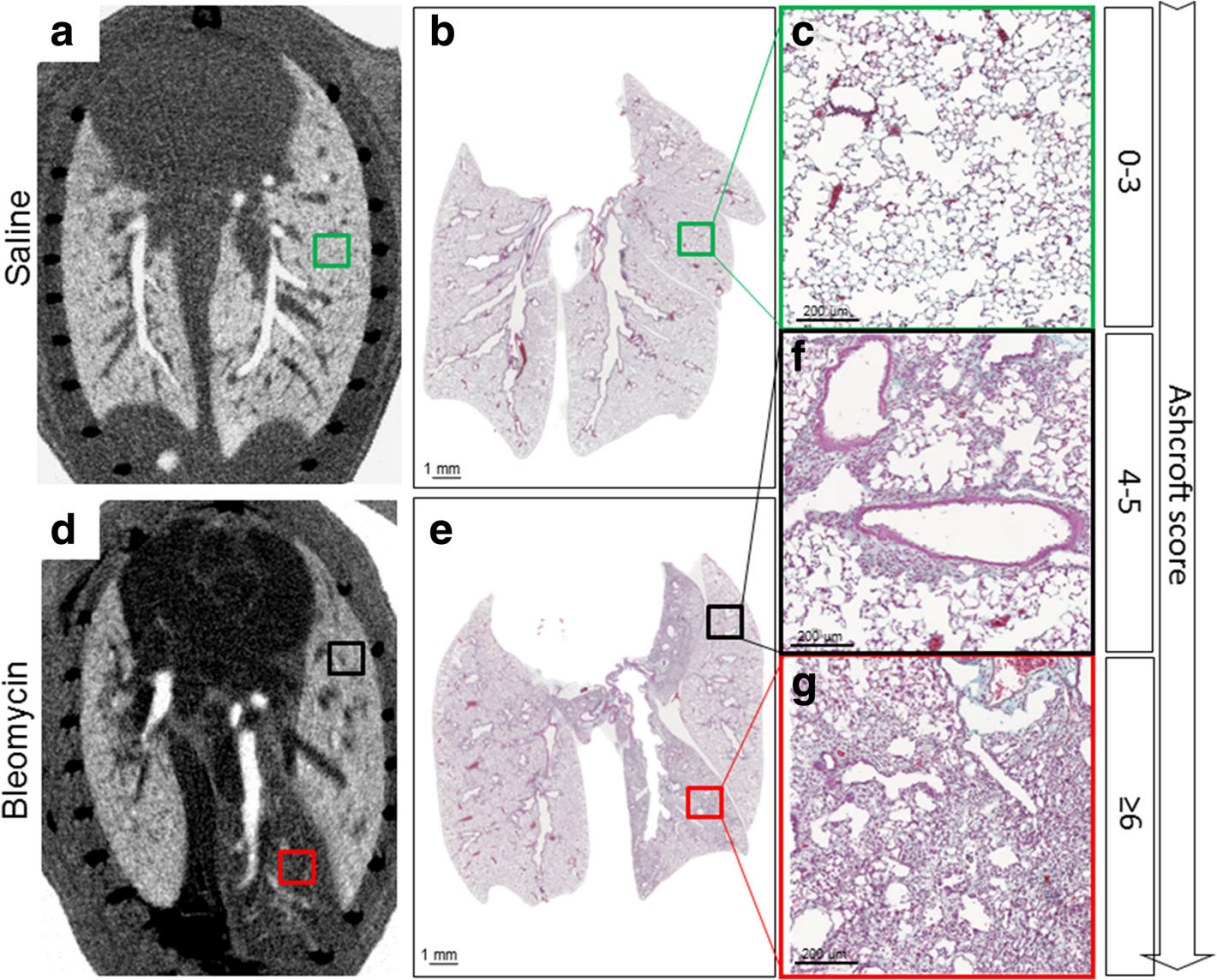
Micro-CT and histology view-matching. **a**, **d** Micro-CT dorsal planes of saline and bleomycin-treated mouse at 14 days fitted with the appropriate histological sections, **b**, **e** (10×) magnification of normal lung parenchyma of saline (**c**), moderately fibrotic (**f**) and heavily fibrotic lung parenchyma of bleomycin-treated mice (**g**). The squares indicate the position and the matching among histology at low magnification and the Micro-CT. The magnification of the *green* and *black squares* (**c**, **f**) indicate normal and moderately fibrotic parenchyma detected by histology and Micro-CT, the *red* one (**g**) indicates the heavily fibrotic lung that appeared as *black* in the Micro-CT view. The respective Ashcroft scores are represented in the *right side* of the figure

To compare the 2-dimensional histological analysis with the Micro-CT images, the lungs were embedded in paraffin in order to maintain their body position and cut along the dorsal plane. Once the serial sections were cut and stained, the corresponding Micro-CT images were identified based on the bronchial tree and the parenchyma structure. For each observation time-point a satisfactory structural matching between the histology slides and the Micro-CT projections was identified. A representative matching between Micro-CT images and lung stained slides obtained from the same individual mice treated either with saline or with bleomycin at 14 days is depicted in Fig. 4a, b, d, and e.

Lung tissue with normal architecture or characterized by mild fibrotic changes, such as alveolar septum thickening, partly enlarged and rarefied alveoli corresponding to an Ashcroft score from 0 to 3 (Fig. 4b and c). The Micro-CT technology was able to visualize and detect the lung parenchyma showing a very well overlapping with histology (Fig. 4a, b and c).

**Fig. 5:**
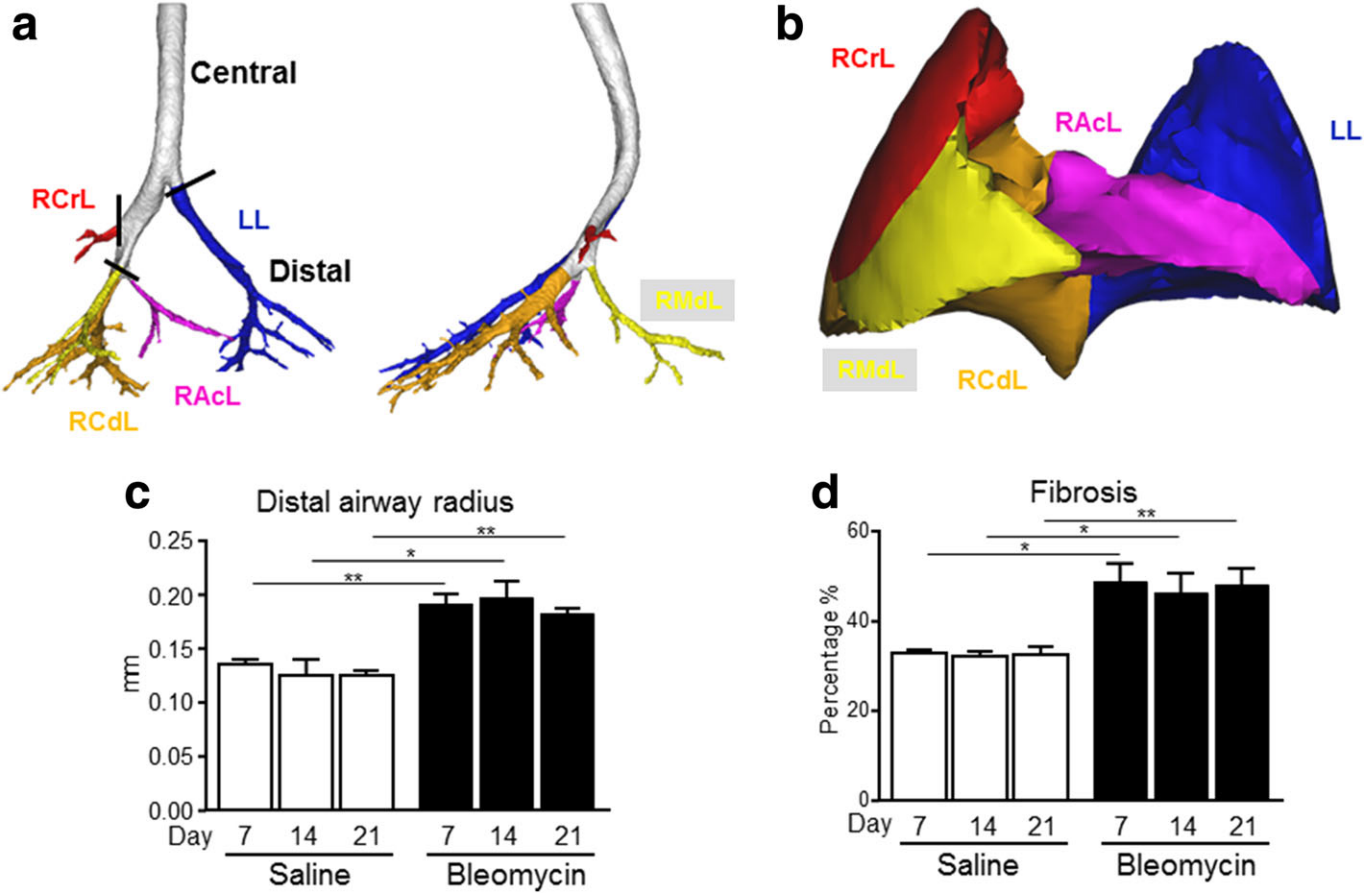
Segmentation and quantification of airways and lung lobes on Micro-CT. Airway models were split into a central and a distal part, that anatomically is the intrapulmonary airway tract, leading to each defined lobe (**a**). Airway radius was evaluated with Micro-CT in the distal part of the airway tree of untreated and treated mice with bleomycin at 7, 14 and 21 day (**c**). Lungs were split into lobes by identification of the fissure lines from the CT scan. Lobes were identified as: Right Cranial Lobe (RCrL), Right Middle Lobe (RMdL), Right Caudal Lobe (RCdL), Right Accessory Lobe (RAcL) and Left Lung (LL) (**b**). Total lung fibrosis was evaluate on Micro-CT of treated mice with saline or bleomycin at 7, 14 and 21 day (**d**). Each point presents the mean ± s.e.m of 5 animals, for a total of 30 mice. Changes were compared to the saline group using one-way ANOVA followed by Dunnett’s t test for multiple comparison. *p < 0.05; **p < 0.01

Single fibrotic masses or confluent conglomerates of substitutive collagen and lesions corresponding to an Ashcroft score of 4–5 (Fig. 4e and f) appeared as darker grey areas in the Micro-CT images, Fig. 4d. In higher density fibrotic areas, classified with an Ashcroft score above 6 (Fig. 4e and g), confluent fibrotic masses were present preventing air access to the alveoli. These regions appeared as black areas in the Micro-CT pictures (Fig. 4d), due to the missing air contrast. In order to quantify the airway radius, airway models were split into a central and a distal part, that anatomically is the intrapulmonary airway tract. Representative pictures of segmented airways and lung lobes from the Micro-CT scan data are shown in Fig. 5a and b, respectively.

The quantification of the airway radius and the percentage of fibrosis from the Micro-CT scan data of saline and bleomycin-treated mice was performed at baseline, 7, 14 and 21 days post-treatment (Fig. 5c and d). A statistical significant increase in fibrosis and airway radius was found for all the time points of observation between the saline and bleomycin-treated mice (Fig. 5c and d), in agreement with histological findings as described in Fig. 2e, f, and g.

**Fig. 6:**
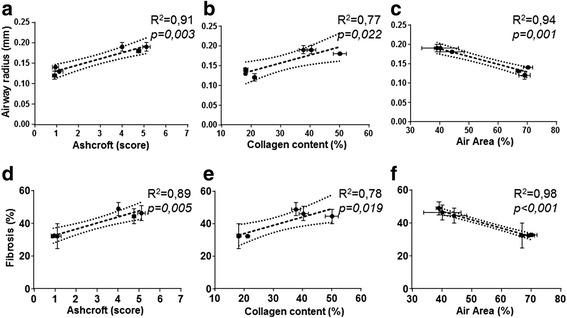
Correlation between Micro-CT and histological parameters. Airway radius (mm) correlation with: Ashcroft score (**a**), Collagen Content % (**b**) and Alveolar Air Area% (**c**) Fibrosis (%) correlation with: Ashcroft score, (**d**), Collagen content % (**e**) and Alveolar Air area % (**b**). Each single dot represents the mean ± s.e.m of 5 animals for a total of 30 mice

The plots in Fig. 6 show the correlation between the two Micro-CT FRI parameters evaluated, fibrosis and distal airway radius, and the three histological measurements: Ashcroft score, alveolar air area and collagen content percentage. Distal airway radius expressed in mm was plotted against Ashcroft score (Fig. 6a), collagen content percentage (Fig. 6b) and alveolar air area percentage (Fig. 6c), for the mean of each group at the different time points. Linear regression analysis showed that airway radius positively correlated with the classical Ashcroft scoring (*r* ^*2*^ = 0.91; *p* = 0.003), (Fig. 6a) and with collagen content percentage (*r* ^*2*^ = 0.77; *p* = 0.022), (Fig. 6b) but negatively with the alveolar air area percentage as expected (*r* ^*2*^ = 0.94; *p* = 0.001), (Fig. 6c).

The same correlation was performed using the FRI fibrosis parameter. Fibrosis percentage was plotted against: Ashcroft score (Fig. 6d), collagen content percentage (Fig. 6e) and alveolar air area percentage (Fig. 6f) for the mean of each group at the different time points. Linear regression analysis showed that fibrosis percentage positively correlated with the classical Ashcroft scoring (*r* ^*2*^ = 0.89; *p* = 0.005) (Fig. 6d) and with collagen content percentage (*r* ^*2*^ = 0.78; *p* = 0.019) (Fig. 6e), but negatively correlated with the alveolar air area percentage as expected (*r* ^*2*^ = 0.98; *p* < *0.001*) (Fig. 6f).

These findings were consistent and demonstrate that FRI parameters from Micro-CT can serve as a reliable indication of the fibrotic changes occurring in the lung parenchyma.

## Discussion

Despite recent advances in therapeutics available for IPF, there is a continued need to find more effective therapies to prevent or reverse lung fibrosis [28]. Moreover, the only treatment proven effective in prolonging survival is lung transplantation with a post-transplantation 5-year survival for IPF patients of approximately 44% [29]. The development of novel therapies relies on how well experimental animal models are able to translate findings to clinical outcomes but there is still much debate around the reliability of current animal models for pulmonary fibrosis [28]. Single bleomycin administration in mice produces fibrotic lesions with an high degree of variability among individual animals and, moreover, the fibrosis tends to resolve spontaneously.

In order to overcome some of the intrinsic animal modelling limitations, we have set up a more robust and reproducible lung fibrosis mouse model that requires a double instillation of bleomycin as detailed in Fig. 1. In vivo imaging in small animal models is a powerful tool to visualize disease progression, to assess their topographical distribution and to qualitatively evaluate the severity in a non-invasive manner. This approach may help to fill the gap between preclinical setting and clinical practice [30].

In the present work we monitored the lung fibrosis in bleomycin-induced mouse model by using Micro-CT technology coupled to classical histology. The major limitation of the conventional assessments of fibrosis relies on invasive ex vivo measurements. These methodologies produce solid and reproducible data, but could be biased from sampling methods. In fact, only a small fraction of the total lung is accounted for the histological evaluation of Ashcroft score, and histomorphometric measurements of collagen content percentage and alveolar air area fraction.

Moreover, mice need to be sacrificed at each time point in order to have a sufficient number for statistical power, precluding the possibility to monitor the disease progression in the same subject. When a mouse is sacrificed and disappears from the experimental setting, a missing information is generated in the biological system. The missing information cannot be retrieved anymore and performing new studies may increase biological variability. Micro-CT seems to be an ideal technology for quantifying pulmonary disease progression lung parenchyma, remodeling and the bronchial tree changes. Micro-CT approach assures that the entire lung is assessed in each animal, while the conventional tools are limited [31].

In this study Micro-CT was used to quantify longitudinally disease progression by evaluating two parameters: airway radius in the distal part of the bronchial tree (Fig. 5a) and fibrosis percentage in the lung parenchyma (Fig. 5b). However, the Micro-CT revealed a limitation on the fibrosis percentage detection at early time points. (Fig. 5d). It is bleomycin treatment induced an inflammatory phase [32] and there is a balance between inflammation and fibrosis at day 7 resulting with slightly overestimation of fibrosis percentage parameter measured by Micro-CT compare to histology. The Micro-CT projections of the lungs presenting mild or moderate degree of fibrosis areas with an Ashcroft score (0–4) found a perfect match with histology (Fig. 4a, b, c, and f). The fibrosis pattern is characterized by a patchy distribution of the fibrotic lesions containing some areas with a high degree of fibrosis with an Ashcroft score (5- > 6), Fig. 4d, e and g. These zones in Micro-CT images appeared darker till almost become black corresponding to an increase in severity of fibrosis, due to the missing contrast from the alveolar air space, limiting the possibility to overlap Micro-CT images with histology. Despite some technical limitations, the correlation plots (Fig. 6) clearly highlight convincing thresholds for each histological parameter and Micro-CT allowed the quantification of distal airway radius and fibrosis (Fig. 5c and d).

An increase of the distal airway radius parameter could be explained by the obliteration of the alveoli and the consequent airflow limitation. This could lead to the airway tree dilation as a compensatory phenomenon, which explains the very good correlation found with Ashcroft score and alveolar air area percentage (Fig. 6a and c) and even though weaker, a significant correlation between airway radius and collagen content (%) was also obtained.

FRI fibrosis parameter increased over the time points of observation. Additionally, the amazing correlation found with alveolar air area percentage (*r* ^*2*^ = 0.98), (Fig. 6f) and with Ashcroft score and collagen content demonstrated that lung parameters from Micro-CT serve as a reliable indicator of the typical lung changes in IPF pathology, involving: airspace fibrosis, alveolar collapse and decrease in the gas-exchange tissue surface.

Moreover, the capability of Micro-CT technology to quantify the fibrosis and the airway radius changes is more likely to reflect directly in the pathologic and also therapeutic changes in the lungs.

Even though the Micro-CT cannot be used as a stand-alone technology at the moment, it seems to be complementary to histology. Micro-CT can provide the 3D model while histology provides more accurate information on the cellular and molecular level.

Our approach with Micro-CT in future drug efficacy studies would be to obtain baseline evaluation of each animal before fibrosis is induced, then confirmation of fibrosis before the treatment is initiated and finally evaluation of potential treatment effects within the therapeutic window.

We believe that this approach represents a smart tool to evaluate drug efficacy and eventually will prove to be superior compared to terminal assessment for drug discovery [31].

Nowadays is well known that drug effects are crucially dependent on timing of compound administration and that the testing of anti-fibrotic agents must be performed during a specific fibrotic phase of the animal model [33]. This limitation can be overcome using Micro-CT scan: all the mice could be screened before entering in the drug treatment and divided according to the severity of the disease. The huge advantage is that each mouse considered as a single subject could be the control of itself; and an interim analysis of drug efficacy could be performed in order to guide and plan in advance the future experiments.

## Conclusions

Drug discovery process is very expensive and unproductive, [34–36] and there is a huge medical need for treating IPF patients. The in vivo study, that recapitulates the pathogenesis, the lesions as well as the effect of a drug on the disease outcome, represents the main obstacle to be overcome during the last steps of development of such molecules.

This combination of better animal models, Micro-CT technology together with histology data allowed a more accurate interpretation of the data obtained in vivo. The possibility to analyze more parameters from the Micro-CT is fundamental in order to fully describe and better understand dynamic processes during IPF disease onset, progression and therapy.

Taken together, our findings underline the importance of the possibility to accurately quantify specific parameters to follow disease progression in a non-invasive way. We strongly believe that Micro-CT applied to the fibrosis research field could allow longitudinally studies reducing the variability and number of animals used and may, therefore, provide a novel tool for understanding pulmonary fibrosis onset, progression and therapy.

## Abbreviations

FRI: Functional respiratory imaging
IPF: Idiopathic pulmonary fibrosis
LL: Left Lung
Micro CT: Micro-Computer Tomography
RAcL: Right Accessory Lobe
RCdL: Right Caudal Lobe
RCrL: Right Cranial Lobe
RMdL: Right Middle Lobe
ROI: Region of interest
